# Serial assessment of computed tomography angiography for pulmonary and systemic arteries using a reduced contrast agent dose for the diagnosis of systemic artery-to-pulmonary artery shunts

**DOI:** 10.1007/s11604-023-01520-0

**Published:** 2023-12-27

**Authors:** Fumiaki Fukamatsu, Akira Yamada, Keiichi Yamada, Tomofumi Nonaka, Takanori Aonuma, Yoshinori Tsukahara, Satoshi Kawakami, Hiroyuki Sasaki, Yasunari Fujinaga

**Affiliations:** 1 Department of Radiology, Shinshu University School of Medicine, 3-1-1 Asahi, Matsumoto, Nagano 390-8621 Japan; 2 Division of Radiology, Shinshu University Hospital, 3-1-1 Asahi, Matsumoto, Nagano 390-8621 Japan

**Keywords:** Computed tomography angiography for pulmonary artery, Computed tomography angiography for systemic artery, Hemoptysis, Reduced contrast agent dose, Systemic artery-to-pulmonary artery shunt

## Abstract

**Purpose:**

To evaluate the diagnostic performance and feasibility of a modified computed tomography (CT) scan protocol, we performed a serial assessment of the computed tomography angiography for pulmonary artery (CTA-P) and systemic artery (CTA-S) (CTA-PS) using a reduced contrast agent dose to diagnose systemic artery-to-pulmonary artery shunts (SPSs).

**Materials and methods:**

Twenty-five patients who underwent multiphase contrast-enhanced chest CT and conventional chest angiography were included. Three image sets (CTA-P, CTA-S, and CTA-PS) were evaluated by two board-certified radiologists. The visualization of the CT image findings associated with SPSs, such as filling defects and enhancement in the pulmonary arteries, was evaluated using a 5-point scale.

**Results:**

The diagnostic performance (sensitivity, specificity, and accuracy) of CT imaging findings associated with SPSs in CTA-P and CTA-PS were as follows: CTA-P, 57.1%, 87.5%, and 62.0%; CTA-PS, 81.0%, 100.0%, and 84.0%. CT findings associated with SPSs in CTA-P were significantly sensitive to the CTA-PS protocol. There were no significant differences between the CTA-S and CTA-PS protocols. The area under the curve (AUC) of the CT imaging findings associated with SPSs in the CTA-P and CTA-PS groups was 0.835 and 0.911, respectively (P = 0.191). The AUC of the CT imaging findings associated with SPSs in CTA-S and CTA-PS were 0.891 and 0.926, respectively (P = 0.373).

**Conclusion:**

CTA-PS using a reduced contrast agent dose protocol could improve the overall diagnostic confidence of SPSs, owing to better visualization of CT imaging findings associated with SPSs compared to individual assessments of CTA-P or CTA-S. Therefore, CTA-PS can be used as an alternative preembolization evaluation modality to conventional angiography in patients with hemoptysis suspected of having SPSs.

## Introduction

Massive hemoptysis, defined as bleeding originating from the lower respiratory tract [1], is a life-threatening thoracic condition. Conservative treatment of massive hemoptysis has a mortality rate of 50–100% [2]. In 90% of cases, the source of massive hemoptysis is bronchial circulation [3]. Bronchial artery embolization is considered the treatment method of choice and the most effective non-surgical treatment for massive and recurrent hemoptysis. Angiographic findings suggestive of the causes of hemoptysis include hypertrophic and tortuous bronchial or non-bronchial systemic arteries, areas of hypervascularity and neovascularity, systemic artery-to-pulmonary arterial or venous shunts, and bronchial artery aneurysms [4–7]. Systemic artery-to-pulmonary artery shunts (SPSs) are anatomical abnormalities that can cause life-threatening recurrent hemoptysis [8]. These abnormal shunts are caused by chronic inflammation, such as bronchiectasis, chronic bronchitis, tuberculosis, and mycotic lung diseases [9, 10]. SPSs have been revealed in 38.9% of cases of life-threatening hemoptysis on angiography [11]. However, angiography is invasive and not recommended as a screening method for SPSs.

In contrast, multidetector row computed tomography (MDCT) is a less invasive screening method for the evaluation of SPSs. The several imaging findings suggestive of SPSs in CT angiography (CTA) are: (1) abnormal enhancement of the responsible pulmonary arterial branches and dilatation of corresponding systemic arterial branches on CT angiography at systemic arterial timing (CTA-S) [12, 13]; and (2) abnormal filling defects in the responsible pulmonary arterial branches on CT angiography at pulmonary arterial timing (CTA-P) [10, 14, 15]. The diagnostic performance of the SPSs for CTA-S has been reported to have high sensitivity and specificity [13].

Although CTA is considered a less invasive screening method for SPSs, it is often difficult to diagnose SPSs confidently differentiating from pulmonary embolism (PE), enhancement unevenness and artifacts using CTA. PE is one of the causes of hemoptysis and is often accompanied by filling defects in the pulmonary arterial branches of CTA-P, mimicking SPSs [15, 16]. In this case, it is important to identify the abnormal enhancement in CTA-S at the same part of the filling defects in the responsible pulmonary arterial branches in CTA-P, which is a characteristic of SPSs, to distinguish SPSs from PE. Therefore, serial assessment of CTA-P and CTA-S (CTA-PS) can be considered to increase diagnostic accuracy and confidence in distinguishing between SPSs and other differential diseases causing hemoptysis. However, CTA-P and CTA-S have not been evaluated simultaneously in diagnosing SPSs due to technical difficulties in serially obtaining CTA-P and CTA-S images with reasonable vascular contrast between the pulmonary and systemic arteries (stagnation of contrast media in pulmonary arteries as few as possible in CTA-S) using a full dose of the contrast agent. Therefore, the synergistic effect of sequential assessment of CTA-P and CTA-S has not been studied so far. The test injection method can be used for CTA-P and CTA-S imaging in addition to the serial scanning method, but there are problems such as contamination by the contrast agent used in the test injection and the laboriousness of the imaging technique.

We considered that abnormal enhancement of pulmonary artery would be easier to identify with lower CT values in the pulmonary arteries and higher CT values in the systemic arteries at CTA-S by increasing bolus nature of the injected contrast agent. Reducing a dose of contrast media, i.e., the injection time is shortened at the same injection rate, would be considered to shorten the contrast duration time in the pulmonary artery, resulting in lower concentrations in the pulmonary artery at CTA-S. Furthermore, increasing injection rate of contrast media would be also considered to contribute to increase bolus nature of injected contrast media and resulted in lower CT values in the pulmonary arteries at CTA-S when CT values in the systemic arteries are high. Low bolus injection protocol using a normal dose of contrast media might result in poor contrast between the pulmonary and systemic arteries at CTA-S because of longer contrast duration time in pulmonary arteries compared to high bolus injection protocol using a reduced contrast media dose.

We hypothesized that CTA-PS using reduced contrast media doses that enhance vascular contrast between the pulmonary and systemic arteries can increase diagnostic accuracy and confidence in distinguishing between SPSs and other differential diseases causing hemoptysis.

In this study, we aimed to evaluate the added value of CTA-PS with a reduced contrast agent dose for the diagnosis of SPSs.

## Material and methods

### Patients

This study was conducted in accordance with the principles of the Declaration of Helsinki. The study protocol was approved by the Institutional Review Board of Shinshu University (Matsumoto, Japan) on August 9, 2022 (approval number: 5588). Informed consent was obtained from participants in the form of an opt-out on the website. We retrospectively reviewed a consecutive database of chest computed tomography (CT) images obtained at our hospital between February 2016 and November 2021. The study population comprised 25 patients (16 men and nine women; age range 32–89 years; mean age, 64 years) who underwent contrast-enhanced chest CT and conventional chest angiography. The purposes of contrast-enhanced chest CT were as follows: screening for hemoptysis (23 patients), anatomic and hemodynamic evaluation of the bronchial arterial aneurysm (1 patient), and posterior mediastinal arteriovenous malformation (1 patient). The final diagnosis was made using conventional chest angiography. The breakdown of diagnosis was as follows: SPSs (21 patients, two of whom had bronchial arterial aneurysms), dilatation of the right bronchial artery (one patient), posterior mediastinal arteriovenous malformation (one patient), and no vascular abnormalities (two patients).

### CT imaging

CT was performed using a multidetector row helical CT scanner (Light Speed VCT or Revolution CT; GE Healthcare, Waukesha, WI, USA). The number of detector rows was 64 in Light Speed VCT and 256 in Revolution CT. The CT scan parameters of precontrast chest CT were as follows: tube voltage = 120 kV, tube current = 550 mA, rotation time = 0.4 s, collimation = 40 mm, beam pitch = 0.984, slice thickness = 1.25 mm, matrix = 512 × 512 in Light Speed VCT; tube voltage = 120 kV, tube current = automatic exposure control (range 100–475 mA), rotation time = 0.28 s, collimation = 80 mm, beam pitch = 0.992, slice thickness = 1.25 mm, matrix = 512 × 512 in Revolution CT. In contrast-enhanced chest CT, tube current = 330 mA, beam pitch = 1.375 in Light Speed VCT; tube current = automatic exposure control (range 100–400 mA), beam pitch = 1.375 in Revolution CT. The other parameters of contast-enhanced chest CT are the same as in precontrast chest CT in two CT scanners, respectively.

An automatic exposure control technique that tailors the mA based on each patient’s body habitus and generates images of diagnostic quality at the minimum possible radiation dose was applied to reduce the radiation dose in the Revolution CT examination [17].

After obtaining a precontrast chest CT image, CTA-PS using a reduced dose (50 mL) of the contrast agent was performed. Various contrast agents were used in this retrospective study. Contrast agents containing 370 mg of iodine/mL (iopamidol; Iopamiron 370; Bayer, Osaka, Japan) or equivalent generic drugs were used; 320 mg of iodine/mL (Ioversol, Optiray 320; Guerbet Japan, Tokyo, Japan) and 300 mg of iodine/mL (Iohexol, Omnipaque 300; GE Healthcare, Tokyo, Japan) were used in one and three patients, respectively. Contrast agents were administered at a rate of 5.0 mL/s; however, a decreased injection rate was used when considered appropriate: 3.5 mL/s in two patients, 4.0 mL/s in one patient, and 4.5 mL/s in one patient (rate range 3.5–5.0 s; mean rate 4.82 s). Under single breath-holding, a multiphase (six or seven phases) contrast-enhanced chest CT was performed to obtain CTA-PS. Owing to the retrospective nature of this clinical study, two CT scan protocols were used. In the seven-phase protocol, each phase was obtained at 6, 11, 16, 21, 26, 31, and 36 s after injection of the contrast agent (before May 2019); the phase at 36 s was not obtained in the six-phase protocol (after May 2019). Furthermore, the radiation dose in the six-phase protocol was adjusted according to diagnostic reference levels (DRLs) 2015 [18].

### Image analysis

**Fig. 1:**
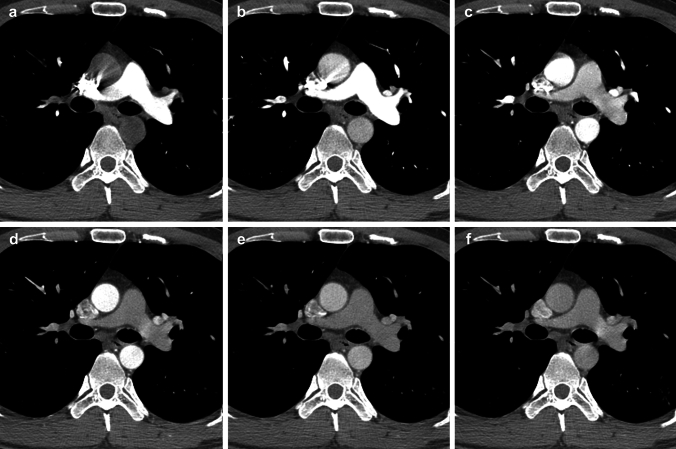
Multiphase contrast-enhanced chest CT images with six phases are shown. Each phase was obtained at **a** 6 s, **b** 11 s, **c** 16 s, **d** 21 s, **e** 26 s, and **f** 31 s after the beginning of contrast agent injection. Images **a** and **b** are included in the image set of CT angiography for the pulmonary artery (CTA-P), and images **c**–**e** in CT angiography for the systemic artery (CTA-S). The serial assessment of CTA-P and CTA-S (CTA-PS) includes images **a**–**e**. Image **f** the phase without difference between pulmonary arteries and thoracic aorta regarding CT values, is excluded from the three image sets. CT, computed tomography

Two board-certified radiologists with 10 and 6 years of experience in chest imaging evaluated the three image sets: CTA-P, CTA-S, and CTA-PS. CTA-P images were defined as phases with higher CT values of the pulmonary arteries than in the thoracic aorta on multiphase contrast-enhanced chest CT; CTA-S images were defined as phases with higher CT values of the thoracic aorta than the pulmonary arteries on multiphase contrast-enhanced chest CT. The CTA-PS image sets included all phases that met the above definition on multiphase contrast-enhanced chest CT. Phases without differences between the pulmonary arteries and thoracic aorta were excluded from the three image sets (Fig. 1). The two observers were not informed of the imaging findings; the final diagnosis was based on conventional chest angiography as a reference standard, which was diagnosed by two board-certified radiologists with 10 and 16 years of experience in angiography, respectively. Any discrepancies in interpretation were resolved through discussion between the two radiologists.

**Fig. 2:**
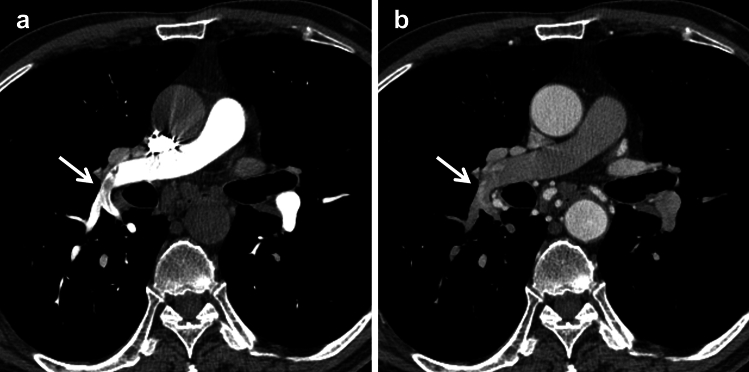
A 65-year-old man with CT image findings associated with systemic artery-to-pulmonary artery shunts (SPSs) **a** CT angiography for the pulmonary artery (CTA-P) shows filling defects in the main pulmonary artery to the inferior pulmonary artery (white arrow). **b** CT angiography for the systemic artery (CTA-S) shows enhancement in the pulmonary artery at the same part of the filling defects in CTA-P (white arrow). *CT* computed tomography

We assessed the visualization capability of CT imaging findings associated with SPSs in each CTA protocol. A visualization of the filling defect and abnormal enhancement of the pulmonary arteries was performed (Fig. 2). Filling defects were evaluated in the CTA-P and CTA-PS image sets, and abnormal enhancement in the pulmonary arteries was evaluated in the CTA-S and CTA-PS image sets. A 5-point scale was used to evaluate CT image findings, and the scale was defined as follows: (1) filling defects or abnormal enhancement in the pulmonary arteries were recognized as not existing; (2) as probably not existing; (3) as difficult to distinguish from enhancement unevenness and artifacts; (4) as probably existing; and (5) as definitely existing. One score was determined for each image set using all phases included in the image set.

### Statistical analysis

The sensitivity, specificity, accuracy, and area under the curve (AUC) of the receiver operating characteristic curve analysis were calculated according to the CT image findings and protocols.

Sensitivity, specificity, and accuracy were calculated considering scores of 4 or 5 as true positives in participants with SPSs and 1 or 2 as true negatives in other diseases except for SPSs. The mean values of the two observers were compared between CTA-P and CTA-PS with regard to filling defects in the pulmonary arteries and between CTA-S and CTA-PS with regard to abnormal enhancement in the pulmonary arteries. Fisher’s exact test was used to evaluate sensitivity, specificity, and accuracy, and DeLong's test was used to determine the AUC. Statistical significance was set at P < 0.05.

The inter-observer variability of the CT image findings for each image set was assessed using Kappa statistics. Kappa values were considered as follows: 0.01–0.20, slight agreement; 0.21–0.40, almost fair; 0.41–0.60, moderate; 0.61–0.80, substantial; and 0.81–1.0, almost perfect.

All statistical analyses were performed using EZR on R Commander Version 2.7-1 (Saitama Medical Center, Jichi Medical University, Saitama, Japan), which is a graphical user interface for R (The R Foundation for Statistical Computing, Vienna, Austria) [19].

## Results

The kappa values of the two observers regarding the two CT image findings for each image set are listed in Table 1. A fair agreement was observed in the CTA-S for assessing the enhancement in the pulmonary arteries. Moderate agreement was observed in CTA-P for the assessment of filling defects in the pulmonary arteries and in CTA-PS for the assessment of enhancement in the pulmonary arteries. Substantial agreement was observed in the CTA-PS for the assessment of filling defects in the pulmonary arteries.

**Table 1:** Kappa values between two observers with regard to two CT image findings for each image set

CT image findings	Kappa values
Filling defects in pulmonary arteries	
CTA-P	0.41
CTA-PS	0.71
Enhancement in pulmonary arteries	
CTA-S	0.38
CTA-PS	0.59

*CTA-P* Computed tomography angiography of the pulmonary artery, *CTA-PS *Serial assessment of computed tomography angiography of the pulmonary and systemic arteries, *CTA-S* Computed tomography angiography of the systemic artery

The sensitivity, specificity, accuracy, and AUC of the two CT imaging findings for each image set are shown in Table 2. The sensitivity, specificity, and accuracy of CTA-PS were higher than those of CTA-P, and the sensitivity and accuracy of CTA-PS were significantly higher than those of CTA-P (P = 0.033 and P = 0.023, respectively).

**Table 2:** Sensitivity, specificity, accuracy, and area under the curve of the two CT image findings for each image set

Diagnostic performance of CT image findings	Filling defects in pulmonary arteries			Enhancement in pulmonary arteries		
	CTA-P	CTA-PS	P value	CTA-S	CTA-PS	P value
Sensitivity	57.1	81.0	0.0338*	76.2	83.3	0.588
Specificity	87.5	100.0	1	87.5	100.0	1
Accuracy	62.0	84.0	0.023*	78.0	86.0	0.436
AUC	0.835	0.911	0.191	0.891	0.926	0.373

The data were expressed as percentages with regard to sensitivity, specificity, and accuracy

*CTA-P* Computed tomography angiography of the pulmonary artery, *CTA-PS* serial assessment of computed tomography angiography of the pulmonary and systemic arteries, *CTA-S* computed tomography angiography of the systemic artery, *AUC* area under the curve

*Statistical significance was set at P < 0.05

High specificity was observed for CTA-S, and high sensitivity, specificity, and accuracy were observed for CTA-PS. The sensitivity, specificity, and accuracy of CTA-PS were higher than those of CTA-S; however, there were no significant differences between the two image sets (P = 0.588, 1, and 0.436, respectively).

Moderate AUC values were observed in the CT imaging findings associated with SPSs on CTA-P and CTA-S. On CT imaging findings associated with SPSs and CTA-PS, a high AUC value was observed. All AUC values were higher for CTA-PS than for CTA-P or CTA-S. The AUC of the CT imaging findings associated with SPSs in the CTA-P and CTA-PS groups were 0.835 and 0.911, respectively (P = 0.191). The AUC of the CT imaging findings associated with SPSs in the CTA-S and CTA-PS groups was 0.891 and 0.926, respectively (P = 0.373). There was no significant difference in the AUC of the CT imaging findings associated with SPSs.

CT doses in this study were (mean ± standard deviation; numbers in parentheses were ranges): the volume CT dose index (CTDIvol) of precontrast chest CT and per one phase of multi-phase contrast-enhanced chest CT were 12.09 ± 3.25 (7.69–15.57) and 6.86 ± 0.97 (5.49–8.07) mGy, respectively; the dose length product (DLP) of precontrast chest CT, per one phase of multi-phases contrast-enhanced chest CT, and Total DLP in one examination were 610.10 ± 172.74 (320.71–967.78), 265.23 ± 23.26 (219.49–304.41), 2360.64 ± 368.14 (1671.40–3096.72) mGy*cm, respectively. Total DLP in one examination of six-phase protocol was 1964.71 ± 220.43 (1671.40–2401.72) mGy*cm.

## Discussion

Our results revealed that CT imaging findings associated with SPSs, the filling defects in pulmonary arterial branches, could be more sensitively detected by the CTA-PS protocol than by the assessment of CTA-P alone. However, there was no significant difference in the detectability of CT imaging findings associated with SPSs between CTA-S and CTA-PS. This indicates that the CT imaging findings associated with SPSs in CTA-P can be more confidently identified with the help of CT imaging findings in CTA-S.

There are only a few case reports on the CT findings of CTA-P and the diagnostic performance of SPSs for CTA-P. Takeuchi et al. reported the diagnostic performance of detecting SPSs using CTA-S alone in 32 patients based on conventional angiography findings. The reported sensitivity and specificity are 83% and 100%, respectively [13]. This is consistent with the findings of this study. Although a direct comparison is not possible due to the difference in the study population, the sensitivity of our proposed method, CTA-PS, using a reduced contrast agent dose, was equal to that of the previous report.

Our proposed method can be used to obtain the CTA-P along with the CTA-S. It is difficult to diagnose PE with CTA-S alone when contrast enhancement is not enough. Furthermore, the SPSs mimicked PE in CTA-P [16]. It has been reported that the combination of filling defects in the pulmonary arteries on CTA-P and enhancement of the pulmonary arteries on CTA-S suggests SPSs [15, 20]. Therefore, obtaining CTA-P and CTA-S simultaneously might contribute to distinguishing SPSs from PE when delayed contrast-enhanced imaging is not sufficient such as the patient with larger circulating blood volume. However, to date, none of the studies have used CTA-PS to detect and diagnose SPSs. In this respect, the clinical significance of this study is high.

**Fig. 3:**
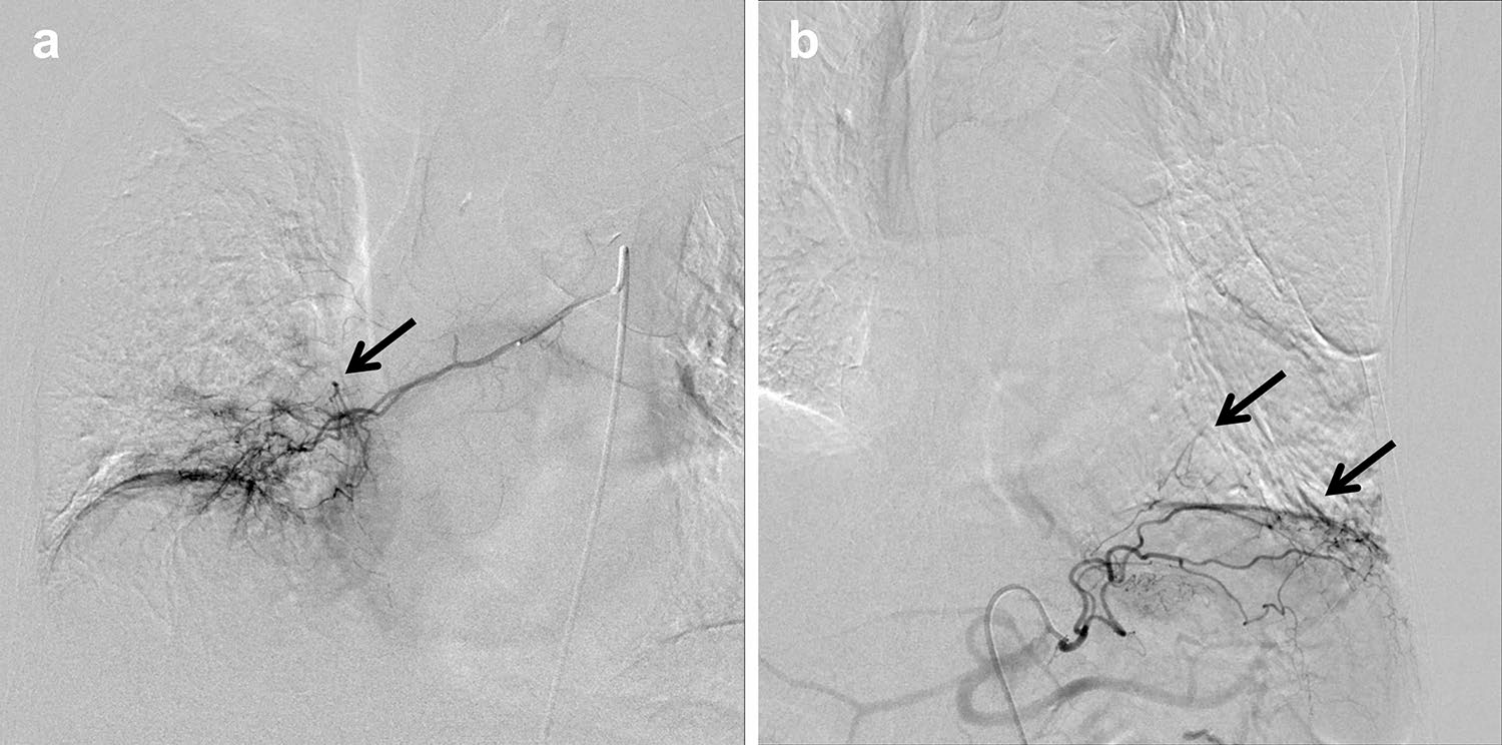
A 73-year-old woman with systemic artery-to-pulmonary artery shunts (SPSs) that could not be detected on multiphase contrast-enhanced chest CT Angiography of the middle and lower lobe branches of the right bronchial artery **a** and left inferior phrenic artery **b** shows microscopic SPSs (black arrows). *CT* computed tomography

However, even if CTA-PS were performed, there were some cases in which observers could not detect the CT image findings associated with SPSs, which was the cause of a decrease in sensitivity, specificity, and accuracy in this study. In these cases, the SPSs were microscopic on conventional angiography; therefore, it was almost impossible to detect associated imaging findings on contrast-enhanced chest CT (Fig. 3). In other words, our proposed CTA-PS method, using a reduced contrast agent dose, can maximize the visualization capability of SPSs using contrast-enhanced CT.

In terms of technical aspects, the radiation dose of the proposed CT scan protocol was within the acceptable range [18]. Several CT protocols for hemoptysis and SPSs, such as CTA using a bolus-tracking technique or 4D CTA, have been reported [8, 21–23]. However, it is often difficult to obtain an optimal CTA because the optimal scan timing for CTA-P and CTA-S from the start of contrast agent injection differs in each case because of the patient’s constitution or circulation function. Compared to CTA-S, CTA-P has a very short optimal scan timing, which is one of the major causes of technical failure in the bolus tracking method. On the other hand, the test injection method predicts the timing of scanning in advance, but there is always a risk that the contrast timing may differ between a small dose of test injection and a large dose injection during the main scan. The CT scan protocol used in this study, CTA-PS, was highly robust against such a scan timing failure. The CT dose per phase of multiphase contrast-enhanced chest CT has been lowered to reduce radiation exposure and minimize image quality degradation. Reducing the total amount of contrast agent while maintaining a high injection rate is also effective in reducing the total scan time and radiation exposure of patients. As the diagnostic performance was equivalent to that of the previous report, CTA images obtained using the proposed CTA-PS protocol could be satisfactory for diagnosing SPSs with a reasonable radiation dose [13]. However, a contrast-enhanced chest CT protocol with fewer phases will be needed to reduce radiation exposure in the future, and methods to predict the optimal scan timing of CTA-P and CTA-S for each case are necessary. Based on the findings of this study, more optimized scanning protocols with reduced contrast load and radiation dose can be realized in the future by combining low-concentration contrast agents with the latest imaging techniques such as dual-energy CT and photon-counting CT [24, 25].

This study had some limitations. First, the number of patients with diseases other than SPSs was small, and there was a difference in the number of patients with these diseases. In many clinical practice cases, conventional angiography is not performed when SPSs are not suspected on multiphase contrast-enhanced chest CT, resulting in fewer cases of diseases other than SPSs. This may have affected our results. Second, conventional angiography of all the arteries that might supply SPSs, such as the bronchial, intercostal, internal thoracic, and branches of the subclavian arteries, was not performed. Third, we tried to reduce the radiation dose of proposed CTA-PS protocol, but the radiation dose of proposed CTA-PS protocol was higher than conventional chest contrast-enhanced CT imaging; therefore, a special care for application of proposed CTA-PS protocol will be needed for the patients with radiation sensitive such as children and pregnant women. It should be necessary to improve proposed CTA-PS protocol to reduce the radiation dose while maintaining the diagnostic performance. However, despite these limitations, our study demonstrated the capability of the proposed CTA-PS protocol as an alternative to conventional angiography for the evaluation of SPSs. In the future, further evaluation of the CTA-PS protocol, including diseases causing hemoptysis other than SPSs in larger populations, is encouraged.

## Conclusion

CTA-PS using a reduced contrast agent dose protocol could improve the overall diagnostic confidence of SPSs, owing to better visualization of CT imaging findings associated with SPSs compared to individual assessments of CTA-P or CTA-S. CTA-PS can be used as an alternative preembolization evaluation modality to conventional angiography in patients with hemoptysis suspected of having SPSs.

## Data Availability

Not applicable.
